# The Packaging Regions of G1-Like PB2 Gene Contribute to Improving the Survival Advantage of Genotype S H9N2 Virus in China

**DOI:** 10.3389/fmicb.2021.655057

**Published:** 2021-04-21

**Authors:** Xiuli Li, Ying Zhao, Shumiao Qiao, Min Gu, Ruyi Gao, Zhichuang Ge, Xiulong Xu, Xiaoquan Wang, Jing Ma, Jiao Hu, Shunlin Hu, Xiaowen Liu, Sujuan Chen, Daxin Peng, Xinan Jiao, Xiufan Liu

**Affiliations:** ^1^Animal Infectious Disease Laboratory, School of Veterinary Medicine, Yangzhou University, Yangzhou, China; ^2^College of Veterinary Medicine, Institute of Comparative Medicine, Yangzhou University, Yangzhou, China; ^3^Yangzhou University Joint International Research Laboratory of Agriculture and Agri-Product Safety of Ministry of Education of China, Institutes of Agricultural Science and Technology Development, Yangzhou University, Yangzhou, China; ^4^Jiangsu Co-innovation Center for Prevention and Control of Important Animal Infectious Diseases and Zoonosis, Yangzhou University, Yangzhou, China; ^5^Jiangsu Key Laboratory of Zoonosis, Yangzhou University, Yangzhou, China

**Keywords:** genotype S H9N2 virus, PB2 gene, packaging region, virus replication, competitive advantage

## Abstract

The genotype S (G57) H9N2 virus, which first emerged in 2007 with the substitution of the G1-like PB2 gene for F98-like ones, has become the predominant genotype in the past 10 years. However, whether this substitution plays a role in the fitness of genotype S H9N2 viruses remains unknown. Comparison of the PB2 genes of F98-like and G1-like viruses revealed a close homology in amino acid sequences but great variations at nucleotide levels. We then determined if the packaging region, a unique sequence in each segment utilized for the assembly of the vRNA into virions, played a role in the fitness of the S genotype. The chimeric H9N2 virus with PB2 segments of the G1-like packaging regions significantly increased viral protein levels and polymerase activity. Substituting the packaging regions in the two terminals of F98-like PB2 with the sequence of G1-like further improved its competitive advantage. Substitution of the packaging regions of F98-like PB2 with those of G1-like sequences increased the infectivity of the chimeric virus in the lungs and brains of chicken at 3 days post infection (dpi) and extended the lengths of virus shedding time. Our study suggests that the packaging regions of the G1-like PB2 gene contribute to improve the survival advantage of the genotype S H9N2 virus in China.

## Introduction

H9N2 viruses have evolved into multiple genotypes (A-W) since its first emergence in China in 1994. These genotypes are divided into five lineages including A/chicken/Beijing/1/1994 (BJ94-like), A/duck/Hong Kong/Y280/1997 (Y280-like), A/quail/HongKong/G1/1997 (G1-like), A/duck/HongKong/Y439/1997 (Y439-like), and A/chicken/Shanghai/F/1998 (F98-like) (Huang et al., 2010; Zhang et al., 2012; Liu et al., 2016; Gu et al., 2017). More than one genotype may circulate simultaneously in one region (Gu et al., 2017). Among a few genotypes prevalent in China before 1999, the BJ94-like series is the predominant one (Gu et al., 2017; Li et al., 2017). Since then, various reassortments have significantly increased the number of the genotypes of H9N2 viruses, whereas the genetic diversity of H9N2 viruses decreased since 2006. The genotype S (G57) H9N2 viruses generated through the reassortment of F98-like viruses by substituting their M and PB2 genes with those of the G1-like emerged first in 2007 and have become predominant in China since 2010s (Gu et al., 2014, 2017). Although the roles of G1-like M and PB2 reassortment in H7N9 and H5Nx viruses have been well characterized (Hao et al., 2019, 2020), the impact of G1-like PB2 on H9N2 virus fitness is yet to be investigated.

The highly selective genome packaging of influenza A viruses relies on complex RNA-RNA or RNA-protein interactions (Liang et al., 2005; Fournier et al., 2012). The specific structures of each vRNA that contribute to the packaging of the vRNA have been identified as packaging signals (Liang et al., 2005; Ozawa et al., 2009; Cobbin et al., 2014; Zhao et al., 2014; Gilbertson et al., 2016). The untranslated regions as well as 300-nt of the coding sequences of PB2 vRNA at both ends are essential for the efficient genome packaging of the WSN virus (Liang et al., 2005, 2008; Muramoto et al., 2006). Recently, stem-loop structures in the terminal packaging sequences in both M and PB2 vRNA are found to be crucial for the infectivity of viruses and the assembly of infectious virion particles (Kobayashi et al., 2016, 2018; Spronken et al., 2017). Our present study aimed at determining if the G1-like PB2 packaging regions contributed to the dominance of genotype S H9N2 viruses. Here we report that substituting the packaging region of F98-like with that of G1-like PB2 led to increased PB2 expression and the production of infectious virus *in vitro* and *in vivo*. Our study unveils a previously unrecognized role of the packaging regions in genotype S H9N2 virus evolution in China.

## Materials and Methods

### Ethics Statement

This study was carried out in strict accordance with the recommendations in the Guide for the Care and Use of Laboratory Animals of the Ministry of Science and Technology of the People’s Republic of China. All animal experiments were approved by the Jiangsu Administrative Committee for Laboratory Animals (Permission number: SYXK-SU-2017-0007), and by the Institutional Biosafety Committee of Yangzhou University and complied with the guidelines of Jiangsu laboratory animal welfare and ethics of Jiangsu Administrative Committee of Laboratory Animals.

### Phylogenetic Analysis of All Segments of H9N2 Viruses

The sequences of PB2 of all H9N2 AIVs isolated between 1996 and 2019 were downloaded from the EpiFlu Database (GISAID): https://www.gisaid.org/registration/terms-of-use/. The Isolate IDs can be found in Supplementary Dataset 1. Each segment was aligned using PhyloSuite, corrected frame shift errors, and then translated to amino acid sequences with mega 5.0. Phylogenetic tree reconstruction and mean group distance were carried out using the Mega 5.0 software with the neighbor-joining method. The robustness of the statistical support for the tree branch was determined by 1,000 bootstrap replicates.

### Minigenome Polymerase Activity Assay

**FIGURE 1:**
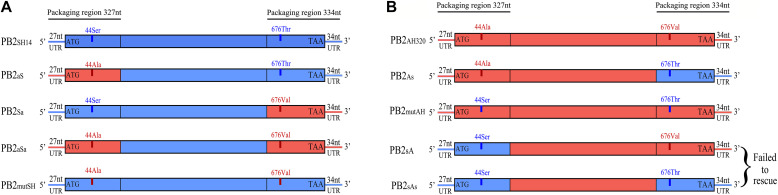
Schematic representation of the chimeric segments. The packaging regions include the untranslated regions (UTRs) and 300 nt of PB2 ORF at 5′ and 3′ terminal. **(A)** The chimeric PB2_*SH14*_ segments (in blue) are flanked by the 5′ or/and 3′ packaging regions of PB2_*AH320*_ (in red), and **(B)** the chimeric PB2_*AH320*_ segments (in red) are flanked by the 5′ or/and 3′ packaging regions of PB2_*SH14*_ (in blue) were constructed. Besides, a segment PB2_*mutSH*_ with S44A and T676V mutation in PB2_*SH14*_ **(A)**, and a segment PB2_*mutAH*_ with A44S and V676T mutation in PB2_*AH320*_ **(B)** were constructed. The recombinant viruses carrying the chimeric segments were generated by reverse genetics in context of AH320 virus.

A set of plasmids were generated on the pHW2000 backbone, carrying each segment of A/Chicken/Anhui/AH320/2016 (genotype S H9N2), F98-like PB2 gene from A/Chicken/Shanghai/14/2001 (H9N2), and chimeric PB2 segments, respectively. 293T cells were transfected with 300 ng firefly luciferase reporter, 30 ng of the control pRL-TK vector, which expresses the Renilla luciferase, and 300 ng each of PB1, PA, NP and PB2 plasmids shown in Figure 1 (Salomon et al., 2006; Marjuki et al., 2007; McAuley et al., 2010). After 48 h, cell extracts were analyzed for luciferase activity by using Dual-Luciferase Reporter assay system (Promega). The results were expressed as the relative activity compared to that in the reaction containing the PB2_*SH*14_ or PB2_*AH*320_ plasmid for each experiment. All results were the means ± SD from three independent experiments.

### Determination of Protein Expression and RNA Synthesis Activity

The protein expression and RNA synthesis were quantified by Western blot and real-time RT-PCR in a four-plasmid expression system or infection conditions, respectively. (i) Triplicate wells of 293T cells were transfected with four plasmids, including PB1, PA, NP, and the chimeric PB2 segment showed in Figure 1, each at 300 ng concentration (Liang et al., 2005, 2008). (ii) Triplicate wells of MDCK cells were infected with each plaque purified virus at a multiplicity of infection of 1 MOI of each virus as described previously (White et al., 2017). Briefly, the infected cells were inoculated at 4°C for 45 min to allow for viruses attachment. Subsequentely, the inoculum was removed, the cells were washed 3 times with cold PBS, and warm opti-MEM was added. After a 3 h incubation period at 37°C, the medium was removed and replaced with the opti-MEM containing 50 mmol ammonium chloride. The absence of TPCK-treated trypsin and the presence of NH4Cl restrict the infection to a single cycle. Forty-eight hours post-transfection/post-infection, the above transfected/or infected cells and the infected supernatants were harvested for subsequent western-blot or RNA extraction. The quantitative real-time RT-PCR was conducted with differences in the primers used for RT and real-time PCR. For the detection of mRNA, vRNA and cRNA, the oligo (dT) primer, the uni-12 primer (5′-AGCAAAAGCAGG-3′) and the uni-13 primer (5′-AGTAGAAACAAGG-3′) were used to generate cDNAs with 500 ng of total RNA from cell sample or 200 ng of total RNA from supernatant sample, respectively. The gene-specific primers for the real-time PCR were 5′-TTGCTCCTTTAATGGTGGC-3′ and 5′-TCCCAGCAGGTCCCTTG-3′ for all PB2 genes, and the probes were VIC-TTGTCCCTCCAGCTACTGG-MGB for the PB2 genes mutated from PB2_*AH*320_ backbone and 5′-FAM-CGGTAGCAGGTGGAACAA-MGB-3′ for the PB2 genes mutated from PB2_*SH*14_ backbone. The PB2 RNA levels shown were as log10 of copy numbers.

### Co-infection of MDCK Cells With Reassortant Viruses

Triplicate wells of MDCK cells were coinfected 1 MOI for each virus. The viruses containing the mutants from PB2_*SH*14_ gene were coinfected with P-WT-AH320 virus, and the viruses containing the mutants from PB2_*AH*320_ gene were coinfected with P-AH320-PB2_*SH*14_ virus. As described above, the medium was replaced with opti-MEM containing 50 mmol NH4CL to ensure a single cycle of viral replication. At 48 hpi, the infected cells and supernatants were collected for RNA extraction, and the RNA levels were detected with the quantitative real-time RT-PCR as described above.

Every experiment was run in triplicate wells and repeated at least twice.

### Animal Experiment

Eight 5 weeks old SPF while leghorn chickens were inoculated intraocularlly with 10^6^ 50% egg infective doses (EID_50_) of each plaque purified mutant viruses. Three chickens were euthanized by CO2 asphyxiation for each group at 3 and 5 dpi, and lungs and brains were collected for virus titration. At 1, 3, 5, and 7 dpi, oropharyngeal and cloacal swabs from five chickens of each group were taken for the detection of viral shedding.

### Statistical Analysis

Statistical analyses were conducted by using SAS software, version 9.2 (SAS Institute. Statistically significant differences between the number of the copies of G1-like PB2 and F98-like PB2 genes were analysed by using Duncan’s multiple range test in ANOVA. Differences were considered significant at *P* < 0.05.

## Results

### Abundant Synonymous Mutations Between G1-Like PB2 and F98-Like PB2

All available PB2 sequences of H9N2 AIV during 1996–2019 were downloaded from the EpiFlu Database (GISAID): https://www.gisaid.org/registration/terms-of-use/. Phylogenetic analysis revealed that the PB2 genes of H9N2 AIV in China are clustered into five independent branches: that is BJ94-like, wild-waterfowl-like, F98-like, G1-like (2006) (which spread prior to 2006) and G1-like that spread since 2007 (Supplementary Figure 1). The mean group distance of each segment is shown as Supplementary Table 1. Nucleotide variations among PB2 nucleotides are much higher than the amino acid sequences of PB2 protein. This suggests that synonymous mutations have occurred during the evolution of the PB2 gene. The conservation of PB2 amino acid sequences may be required for maintaining the structure and function of PB2 polymerase (Zhao et al., 2011).

### Generation of Reassortant Viruses by Reverse Genetics

The coding and non-coding regions in the two ends of the PB2 gene are involved in genome incorporation into the virion of WSN virus (Liang et al., 2005, 2008; Muramoto et al., 2006). To investigate the effect of PB2 nucleotide variations on the adaptability of H9N2 virus, we constructed chimeric PB2 segments by exchanging the packaging regions of PB2_*SH*14_ (F98-like) and PB2_*AH*320_ (G1-like) genes (Figure 1). There are two amino acid differences at position 44 and 676 in the region of the packaging region between PB2_*SH*14_ and PB2_*AH*320_ genes. We constructed a PB2_*mutSH*_ segment with S44A and T676V mutation in PB2_*SH*14_ (Figure 1A), and a PB2_*mutAH*_ segment with A44S and V676T mutation in PB2_*AH*320_ gene (Figure 1B). Eight reassortant H9N2 viruses (AH320-PB2_*SH*14_, AH320-PB2_*aS*_, AH320-PB2_*Sa*_, AH320-PB2_*aSa*_, AH320-PB2_*mutSH*_, WT-AH320, AH320-PB2_*As*_, and AH320-PB2_*mutAH*_) harboring the mutant PB2 segment mentioned above were successfully rescued in the background of AH320 virus. However, the attempt to generate AH320-PB2_*sA*_ and AH320-PB2_*sAs*_ reassortants was not successful. By serial plaque purification, P-AH320-PB2_*SH*14_, P-AH320-PB2_*aS*_, P-AH320-PB2_*Sa*_, P-AH320-PB2_*aSa*_, P-AH320-PB2_*mutSH*_, P-WT-AH320, P-AH320-PB2_*As*_, and P-AH320-PB2_*mutAH*_ viruses were obtained from the rescued viruses.

### Chimeric PB2 Segments Carrying G1-Like Packaging Regions Increase PB2 Expression and Polymerase Activity

**FIGURE 2:**
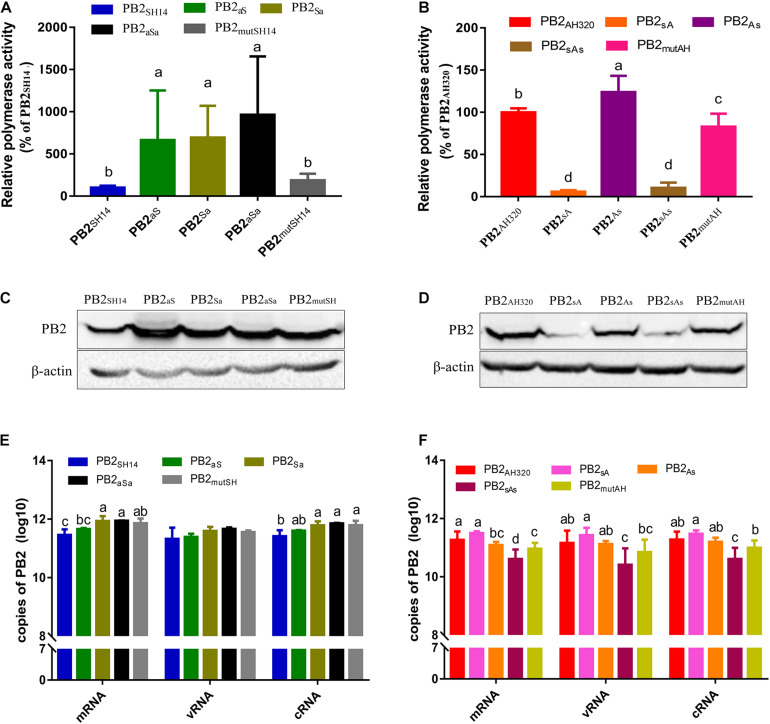
The impact of packaging sequences on relative polymerase activity, protein expression and RNA synthesis of PB2 genes. **(A,B)** Polymerase activity was assessed using a luciferase dual-reporter system. Results were presented as the means ± SD from three independent transfections, and were normalized to transfection efficiency and to the activity of the minigenome containing the PB2_*SH*14_ **(A)** or PB2_*AH*320_ **(B)** within each experiment. **(C–F)** Protein expression **(C,D)** and RNA synthesis **(E,F)** of chimeric PB2 genes. 293T cells were transfected with plasmids expressing PB1, PA, NP and each chimeric PB2 segment. At 48 h post-transfection, the protein expression and RNA synthesis of each PB2 was analyzed by western blotting and real-time RT-QPCR, respectively. Data are represented as mean ± SD (*N* = 3). The statistically significant differences were analyzed by ANOVA. Labeled means in a row without a common letter differ, *P* < 0.05.

To determine the relative contribution of PB2 vRNA sequences to the prevalence of genotype S H9N2 viruses, we constructed chimeric PB2 segments by exchanging the packaging regions of PB2_*SH*14_ and PB2_*AH*320_ genes (Figure 1). We then examined the polymerase activity of these mutant PB2 genes using a minigenome system in a constant AH320 background. As shown in Figure 2A, substituting 5′ or/and 3′ terminal packaging regions of PB2_*SH*14_ with PB2_*AH*320_ sequences increased the viral polymerase activity at different levels. However, the amino acid mutation of PB2_*SH*14_ in 44 and 676 positions did not significantly affect the polymerase activity in the AH320 background. Correspondingly, the RNA and protein levels of the chimeric segments were significantly elevated in most mutant segments based on PB2_*SH*14_ (Figures 2C,E). However, replacing the packaging region at the 5′ end of PB2_*SH*14_ with the G1-like sequence of PB2_*AH*320_ had limited effect on its RNA synthesis (Figure 2E, PB2_*aS*_).

The G1-like packaging regions of PB2_*AH*320_ were reciprocally substituted by the F98-like sequences (Figure 1B). Except PB2_*As*_, all other mutant PB2 genes reduced viral polymerase activity. PB2_*sA*_ and PB2_*sAs*_ decreased viral polymerase activity most effectively (Figure 2B). The levels of PB2_*As*_ and PB2_*mutAH*_ proteins did not significantly decrease. However, the levels of PB2_*sA*_ and PB2_*sAs*_ proteins were much lower than their parental PB2_*AH*320_ gene (Figure 2D). Failure to produce reassortant viruses AH320-PB2_*sA*_ and AH320-PB2_*sAs*_ was likely due to the poor expression of their proteins. Besides, some studies reported that the secondary structure of AIV segments rather than their wild type nucleotide sequences may play a more important role in the incorporation of vRNA process (Dadonaite et al., 2019). The vRNA structure alteration of PB2 segment caused by PB2sA and PB2sAs may disturb the redundant and plastic network of RNA-RNA and potentially RNA-nucleoprotein interactions which are believed to be crucial for AIV genome packaging (Liang et al., 2005; Muramoto et al., 2006; Fournier et al., 2012; Bolte et al., 2019). The substitution of the packaging regions in PB2_*AH*320_ only slightly decreased RNA levels (Figure 2F). Consistently, the sequence exchange at 5′ end of PB2_*AH*320_ (PB2_*sA*_ segment) had little impact on RNA synthesis, either.

The observation that mRNA synthesis level of several mutants was inconsistent with their protein expression may be related to the post-transcriptional processing, regulation and translation of PB2 gene (Chen et al., 2002; Griffin et al., 2002).

### The Packaging Regions of G1-Like PB2 Improves the Production of Infectious Virus Particles

It’s reported that non-infectious viruses are usually associated with a decrease in PFU/HAU or TCID50/HAU ratio (Xue et al., 2016; Bolte et al., 2019). In our study, the virus titers and their infectious particles in the rescued virus stock were measured by the HA test [hemagglutination units (HAU)] and the TCID_50_ values, respectively (Carter and Mahy, 1982a,b; Xue et al., 2016). The percent of infectious particles were calculated based on the ratio of TCID_50_ to HAU. As shown in Table 1, the mutant virus of AH320-PB2_*SH*14_ gave a lowest TCID_50_/HAU ratio of 87 among the reassortant viruses. The TCID_50_/HAU ratio of the mutant viruses that replaced the packaging regions of PB2_*SH*14_ with G1-like sequences of PB2_*AH*320_ gene were improved to the range from 405 to 873. In contrast, the TCID_50_/HAU ratios of mutant viruses AH320-PB2_*As*_ and AH320-PB2_*mutAH*_ were 582 and 8,734, which were much lower than that of their parental WT-AH320 virus.

**TABLE 1:** The infectivity titer (TCID50/100 μl) and Total titer (HA/25 μl) change between the viruses allantoic fluid before and after purifying by series plaque.

	Allantoic fluid of rescued viruses			Allantoic fluid of plaque purified viruses		
		
Viruses	Infectivity titer (TCID_50_/100 μl)	Total titer (HA/25 μl)	TCID_50_/HAU	Infectivity titer (TCID_50_/100 μl)	Total titer (HA/25 μl)	TCID_50_/HAU
WT-AH320	6.5	8	12,352	8.5	11	154,408
AH320-PB2_As_	5.625	9.5	582	8.5	11	154,408
AH320-PB2_mutAH_	6.5	8.5	8,734	8.33	10.5	147,633
AH320-PB2_SH14_	4.5	8.5	87	8.0	11	48,828
AH320-PB2_Sa_	5.167	8.5	405	7.67	10	45,677
AH320-PB2_aS_	5.33	8	835	8.5	11	154,408
AH320-PB2_aSa_	5	7	781	7.5	9	61,763
AH320-PB2_mutSH_	5.5	8.5	873	8	11	48,828

These data demonstrated that the rescued viruses contained non-infectious viral particles, and the viruses harboring the packaging regions of F/98-like PB2 gene produced non-infectious viral particles more abundantly than that of G1-like packaging regions. To minimize contamination of the rescued virus, all the viruses were purified by a series of plaque experiments (Xue et al., 2016). As expected, the TCID50/HAU ratio was elevated (Table 1), indicating the increase of infectious viral particles in rescued viruses.

**FIGURE 3:**
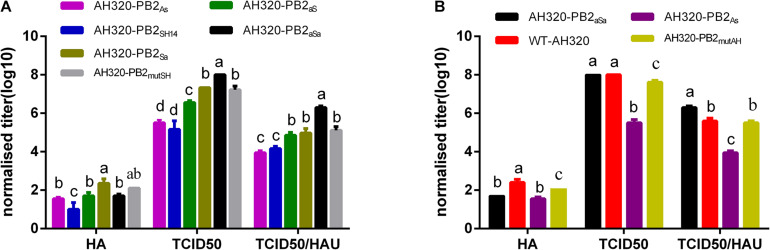
The impact of packaging sequences of PB2 gene on the production of infectious virus particles (the viruses used in the experiments were not purified by plaques). MDCK cells were infected with viruses at 0.1 MOI. At 48 hpi, the HA, TCID50 and TCID50/HAU ratio of each mutant PB2 gene derived from PB2_*SH14*_ **(A)** or PB2_*AH320*_ **(B)** backbone in supernatant were tested. Data are represented as mean ± SD (*N* = 3). The statistically significant differences were analyzed by ANOVA. Labeled means in a row without a common letter differ, *P* < 0.05.

To further explore the effects of PB2 packaging regions on the production of virus particles, the TCID_50_/HAU ratio of the mutant virus in the conditioned media of MDCK cells at 48 hpi were calculated. Compared to AH320-PB2_*SH*14_ virus, the number of progeny virus particles and the proportion of infectious virus particles were improved by replacing the packaging regions of PB2_*SH*14_ with that of PB2_*AH*320_ gene (Figure 3A). The replacement of the packaging region at 3′ end of PB2_*SH*14_ had a greater impact on the generation of virus particles than that at the 5′ end. Replacing the packaging regions of PB2_*SH*14_ at both ends increased the production of infectious virus particles to the levels equivalent to the WT-AH320 virus (Figure 3B, AH320-PB2_*aSa*_). Substitution of 3′-terminal packaging region of the PB2_*AH*320_ gene reduced the proportion of infectious virus particles to a level similarly to that of AH320-PB2_*SH*14_ virus (Figure 3A, AH320-PB2_*As*_). While amino acid substitutions at 44 and 676 positions in PB2_*SH*14_ increased the production of infectious virus (Figure 3A), but had mild effect in PB2_*AH*320_ (Figure 3B).

### The Packaging Regions of G1-like PB2 Gene Increase H9N2 Virus Replication

**FIGURE 4:**
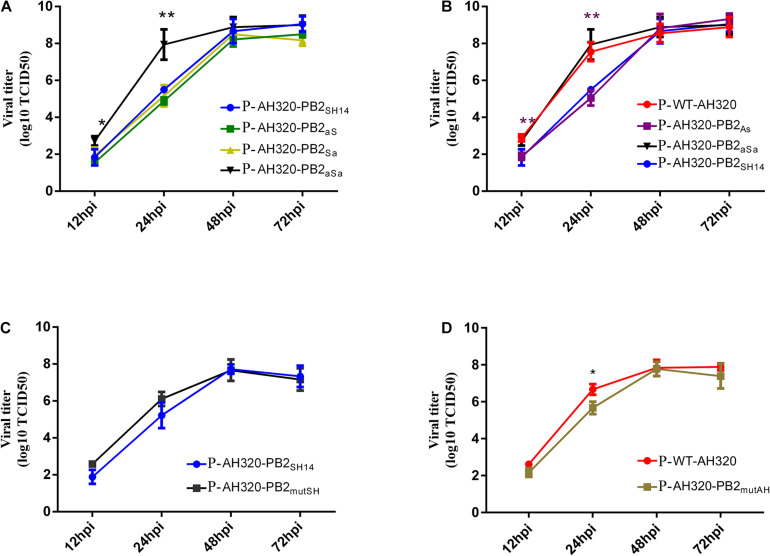
Multiple-cycle growth curves of plaque purified H9N2 viruses. The replication abilities of each virus in MDCK cells was measured by infecting cells at an MOI of 0.1. Virus titers in supernatant were determined in MDCK cells at indicated time points. **(A)** The growth kinetics of the mutant viruses that replaced the packaging regions of PB2_*SH14*_ at either or both ends with that of PB2_*AH320*_. **(B)** The growth kinetics of the mutant viruses that replaced the packaging regions of PB2_*AH320*_ at either or both ends with that of PB2_*SH14*_. **(C,D)** The effect of amino acids at position 44 and 676 of PB2 gene on the replication of H9N2 viruses. Data are represented as mean ± SD (N = 3). The statistically significant differences were analyzed by ANOVA compared with P-AH320-PB2_*SH*14_ or P-WT-AH320 virus (*P < 0.05; **P < 0.01).

We next determined the growth curve of plaque-purified viruses. As shown in Figure 4A, P-AH320-PB2_*aS*_ and P-AH320-PB2_*Sa*_ viruses that substituted the packaging region of PB2_*SH14*_ at either end replicated similarly fast as did P-AH320-PB2_*SH*14_ (Figure 4A). While replacing the packaging regions of PB2_*SH*14_ at both ends with that of PB2_*AH*320_ (P-AH320-PB2_*aSa*_ virus) increased virus replication to the level comparable to P-WT-AH320 virus (Figures 4A,B). Reciprocally, substitution of the packaging region at 3′ end of PB2_*AH*320_ lowered the rate of P-AH320-PB2_*As*_ virus replication to a level similar to P-AH320-PB2_*SH*14_ (Figure 4B). However, alteration of amino acids at position 44 and 676 of either PB2_*SH*14_ or PB2_*AH*320_ had little effect on the replication of H9N2 viruses (Figures 4C,D). Similar observations were made with the use of CEF cells as well as in MDCK cells (Supplementary Figure 2).

### Packaging Regions of PB2 Are Involved in Competitive Advantage in Co-infection

**FIGURE 5:**
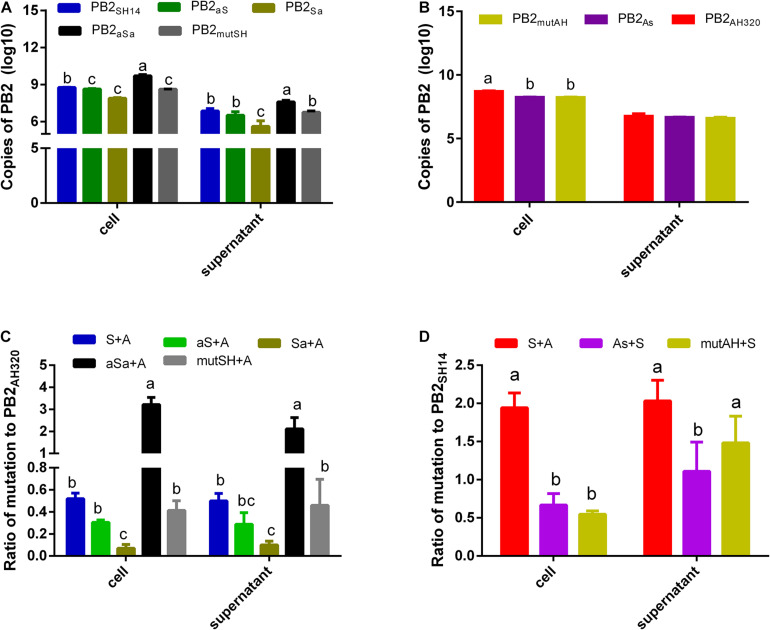
MDCK cells were infected **(A,B)** or coinfected **(C,D)** with plaque purified viruses at 1 MOI. And an inhibitor of endosome acidification, ammonium chloride was added to restrict the infection to a single cycle. At 48 hpi, the copy number of each PB2 gene in infected cells and supernatants was detected by real-time RT-QPCR. And the ratios to PB2_*AH320*_/PB2_*SH14*_ of each mutant PB2 gene derived from PB2_*SH14*_/PB2_*AH320*_ backbone were calculated in coinfection groups **(C,D)**. S, P-AH320-PB2_*SH*14_; aS, P-AH320-PB2_*aS*_; Sa, P-AH320-PB2_*Sa*_; aSa, P-AH320-PB2_*aSa*_; mutSH, P-AH320-PB2_*mutSH*_; A, P-WT-AH320; As, P-AH320-PB2_*As*_; mutAH, P-AH320-PB2_*mutAH*_ in **(C,D)**. Data represent the mean ± SD from six independent infections. The statistically significant differences were analyzed by ANOVA. Labeled means in a row without a common letter differ, *P* < 0.05.

The copy number of PB2_*SH*14_ gene was significantly higher than that of PB2_*aSa*_ vRNA but lower than that of PB2_*Sa*_ vRNA in the cell lysates and conditioned media of MDCK cells infected with 1 MOI of reassortant virus (Figure 5A). While vRNA of PB2_*aS*_ and PB2_*mutSH*_ accumulated comparable copies with that of PB2_*SH*14_ gene in cells and supernatants (Figure 5A). Substitution of packaging sequences at 3′ end or amino acid mutation at 44 and 676 positions in PB2_*AH*320_ resulted in a moderate reduction of the copy number of PB2 vRNA in infected cells, but had little impact on the number of PB2 copies in the infected supernatant (Figure 5B).

When co-infected with P-WT-AH320, the ratio of PB2_*SH*14_/PB2_*AH*320_ copy number was approximately 0.52 in co-infected cells and 0.50 in the conditioned media. Replacing the packaging regions of PB2_*SH*14_ at two ends with that of PB2_*AH*320_ sequences (P-AH320-PB2_*aSa*_ virus) further increased this ratio in the co-infected cells and conditioned media (Figure 5C). In contrast, the ratios of PB2_*aS*_/PB2_*AH*320_ and PB2_*Sa*_/PB2_*AH*320_ copy number decreased in both the virus-infected cells and conditioned media. Amino acid alterations at position 44 and 676 in PB2_*SH*14_ had little effect on its competition advantage. Reciprocally, replacing the 3′-terminal packaging region of PB2_*AH*320_ with the F98-like sequences from PB2_*SH*14_ (P-AH320-PB2_*As*_) reduced PB2_*As*_/PB2_*SH*14_ ratio in the co-infected cells and conditioned media (Figure 5D). Substitution of two amino acids in PB2_*AH*320_ decreased the number of PB2_*mutAH*_ copy in the cells but not in the conditioned media.

### The Packaging Regions of G1-Like PB2 Extend the Lengths of Virus Shedding

**FIGURE 6:**
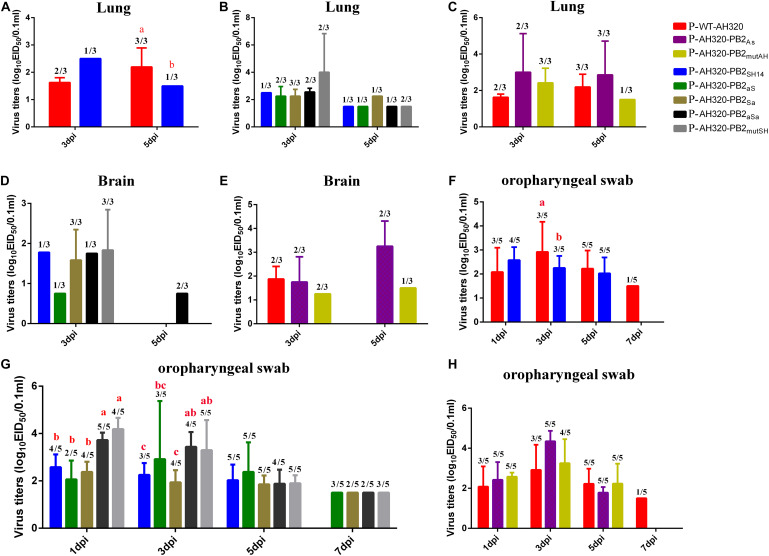
Virus titers of plaque purified viruses P-WT-AH320, P-AH320-PB2_*As*_, PAH320-PB2_*mutAH*_, P-AH320-PB2_*SH*14_, P-AH320-PB2_*aS*_, P-AH320-PB2_*Sa*_, P-AH320-PB2_*aSa*_, and P-AH320-PB2_*mutSH*_ recovered from chicken lungs **(A–C)**, brains **(D,E)**, and oropharyngeal swabs **(F–H)**. Eight 5 weeks old SPF while leghorn chickens were inoculated by intraocularlly with 10^6^ EID_50_ of plaque purified mutant viruses. At 1, 3, 5, and 7 dpi, oropharyngeal swabs from five chickens of each group were taken for the detection of viral shedding, and lungs and kidneys were harvested from three chickens per group at 3 and 5 dpi for virus titration. Virus titers are means ± standard deviations. Statistical significance was based on two-way ANOVA. Labeled means in a row without a common letter differ, *P* < 0.05.

The P-WT-AH320 virus produced significantly higher titers in lungs than did the reassortant virus carrying the PB2_*SH*14_ segment (P-AH320-PB2_*SH*14_) at 5 dpi (Figure 6A). Viruses were detected in the lungs (Figure 6B) and brain (Figure 6D) in one of three chickens infected with the P-AH320-PB2_*SH14*_ virus at 3 and 5 dpi. The number of chickens tested positive in lungs for the mutant virus that replacing the packaging regions of PB2_*SH*14_ with that of PB2_*AH*320_ gene increased at 3 dpi (Figure 6B). And a similar increased number of chickens tested positive for the mutant virus in brains were observed (Figure 6D). However, substituting the packaging regions of PB2_*AH*320_ with the F98-like sequences had little influence on the viral load in lungs or brains (Figures 6C,E).

At 7 dpi, the P-WT-AH320 virus could still be detected in one of five chickens from the oropharyngeal swabs, while the P-AH320-PB2_*SH14*_ virus shedding from the trachea only lasted for 5 days (Figure 6F). Virus loads were significantly higher in the trachea of chickens infected with P-AH320-PB2_*aSa*_, and P-AH320-PB2_*mutSH*_ at 1 and 3 dpi than their parental P-AH320-PB2_SH14_ virus (Figure 6G). All mutant viruses remained detectable in oropharyngeal swabs at 7 dpi (Figure 6G). On the contrary, the tracheal shedding of the mutants P-AH320-PB2_*As*_ and P-AH320-PB2_*mutAH*_ was curtailed to 5 days (Figure 6H).

Viruses were detectable in cloacal swabs from chickens infected with the P-WT-AH320 virus at 1, 3, 5 dpi but not detectable in those infected with the P-AH320-PB2_*SH14*_ virus until at 5 dpi (Table 2). However, viruses were detected at 1, 3, and 5 dpi in cloacal swabs from chickens infected with the viruses replacing the packaging regions of PB2_*SH*14_ with the G1-like sequences of PB2_*AH*320_ (the AH320-PB2_*aS*_, AH320-PB2_*Sa*_, AH320-PB2_*aSa*_, and AH320-PB2_*mutSH*_ viruses, Table 2).

**TABLE 2:** Virus shedding for the inoculated chickens infected with the reassortant H9N2 viruses.

Strains	1 dpi	3 dpi	5 dpi	7 dpi
	
	CL	CL	CL	CL
WT-AH320	1.5 ± 0 (1/5)	1.50 ± 0 (1/5)	1.50 ± 0 (3/5)	ND
AH320-PB2_As_	1.25 ± 0.35 (3/5)	2.00 ± 0.50 (4/5)	2.19 ± 0.41 (4/5)	ND
AH320-PB2_mutAH_	ND	2.00 ± 0.54 (3/5)	2.38 ± 0.88 (4/5)	ND
AH320-PB2_SH14_	ND	ND	1.5 ± 0 (2/5)	ND
AH320-PB2_aS_	1.13 ± 0.38 (2/5)	1.50 ± 0 (3/5)	1.56 ± 0.11 (4/5)	ND
AH320-PB2_Sa_	ND	1.50 ± 0 (2/5)	ND	ND
AH320-PB2_aSa_	ND	1.50 ± 0 (2/5)	1.69 ± 0.32 (4/5)	ND
AH320-PB2_mutSH_	ND	3.33 ± 0.59 (3/5)	2.33 ± 1.01 (3/5)	ND

**Five 5 weeks old SPF chickens were inoculated with 10^6^ EID50 of the H9N2 viruses in a volume of 0.1 ml, respectively. At 1, 3, 5, and 7 dpi, cloacal swabs were taken and suspended in 1 ml of PBS, for the detection of virus shedding in eggs. dpi, day post-inoculation; CL, cloacal swab.**

## Discussion

Codon usage is crucial for translational elongation efficiency and protein folding (Sabi and Tuller, 2014). Synonymous codons are biasedly used by a variety of microbes (Sharp et al., 1988; Plotkin and Kudla, 2011). Our present study shows that the amino acid sequences of the PB2 gene among the five independent branches were relatively conserved, but their nucleotide sequences were variable (Supplementary Table 1). This suggests the presence of a large number of synonymous codons in the PB2 gene. Nucleotide variations between F98-like and G1-like PB2 genes are likely to produce different RNA structures that affect mRNA splicing, protein translation, vRNA packaging, and influenza A virus replication (Kobayashi et al., 2016; Spronken et al., 2017; Baranovskaya et al., 2019).

There is growing evidence that packaging signals play important roles in virus replication, genome incorporation, and genetic reassortment of influenza A virus (Gao and Palese, 2009; Essere et al., 2013; Gerber et al., 2014). Substitutions of the packaging regions of PB2_*SH*14_ and PB2_*AH*320_ genes significantly affected the protein expression and RNA synthesis of the PB2 segment. Moreover, the proportion of infectious virus particles were elevated by substituting the packaging regions of F98-like PB2 gene with the G1-like sequences relative to their parental AH320-PB2_*SH*14_ virus.

Our study also showed that replacing the both ends packaging regions of PB2_*SH*14_ with the PB2_*AH*320_ sequences enhanced its copy number ratio in co-infections, whereas, replacing 3′ or 5′ end packaging region decreased this ratio. One explanation is that different nucleotide sequences in the packaging regions between PB2_*SH*14_ and PB2_*AH*320_ leads to different RNA structures or interactions among segments. Previous studies have shown that different segments of influenza virus acquire unique RNA conformations and form RNA interactions between intra- and intersegment inside virions (Dadonaite et al., 2019). The variation of vRNA structure may have a great impact on the genome packaging, replication and reassortment of influenza virus (Fournier et al., 2012; Gavazzi et al., 2013; Gerber et al., 2014; Shafiuddin and Boon, 2019). Another explanation is that the internal coding regions of PB2 harbor an unknown packaging sequence, as suggested by recent studies showing that the internal coding regions of the PB1 gene also play an important role in the packaging of influenza A virus genome (Liang et al., 2005; Muramoto et al., 2006; Gilbertson et al., 2016). In support of this notion, we found that amino acid alterations at position 44 and 676 of PB2_*AH*320_ decreased the ratio of PB2_*mutAH*_/PB2_*SH*14_ copy number in co-infected cells (Figure 5D) but did not affect this ratio in the viruses in the conditioned media. This result indicates that in complex coinfection conditions, the amino acid mutation reduced vRNA replication of PB2_*mutAH*_, but did not influence its competitive advantage at incorporation stage.

*In vitro* cell culture experiments revealed that the packaging regions of G1-like PB2 increased virus replication. However, the packaging regions of G1-like PB2 only modestly enhanced virus replication in chickens but pronouncing extended the length of virus shedding time and the odds of virus detection in trachea and cloacae. These results suggest that viruses harboring PB2 genes with the G1-like packaging sequences produce more infectious virus particles, leading to the dominance of H9N2 virus in circulation.

Influenza virus reassortment is controlled by many factors such as compatibility among polymerase subunits and segments mismatch (Marshall et al., 2013; White and Lowen, 2018). Most reassortment events lead to incompatibilities at protein and vRNA levels, resulting in the production of defective virions (White and Lowen, 2018). The persistent epidemics of genotype S H9N2 virus in China implies the high compatibility among its vRNA segments.

In summary, our study has demonstrated that the packaging sequences of G1-like PB2 enhanced the production of infectious viral particles of H9N2 virus *in vitro* and *in vivo*. Replacing the packaging regions of F98-like PB2 at both ends with the G1-like sequences further enhance the competitive advantages of virus replication. Our study suggests that H9N2 viruses acquire replication advantages by optimizing their PB2 nucleotide sequences.

## Data Availability Statement

Publicly available datasets were analyzed in this study. The data presented in the study are deposited in the EpiFlu Database (GISAID): https://www.gisaid.org/registration/terms-of-use/. The isolate IDs can be found in Supplementary Dataset 1. The datasets generated and analyzed during this current study are available from the corresponding author.

## Ethics Statement

The animal study was reviewed and approved by the Jiangsu Administrative Committee for Laboratory Animals (Permission number: SYXK-SU-2017-0007), the Institutional Biosafety Committee of Yangzhou University, and Jiangsu laboratory animal welfare and ethics of Jiangsu Administrative Committee of Laboratory Animals.

## Author Contributions

XLL and MG designed this study. XLL, YZ, SQ, RG, ZG, and JM performed the experiments. XLL, MG, XX, XW, JH, SH, XWL, SC, DP, XJ, and XFL drafted and revised the manuscript. All authors read and approved the final manuscript.

## Conflict of Interest

The authors declare that the research was conducted in the absence of any commercial or financial relationships that could be construed as a potential conflict of interest.

## Supplementary Material

Supplementary Figure 1 Phylogenetic trees of PB2 gene segments of the H9N2 viruses during 1996–2019 isolated in China.

Supplementary Figure 2 Multiple-cycle growth curves of plaque purified H9N2 viruses in CEF cells. The replication abilities of each virus in CEF cells was measured by infecting cells at an MOI of 0.1. Virus titers in supernatant were determined in CEF cells at indicated time points. Data are represented as mean ± SD (*N* = 3). The statistically significant differences were analyzed by ANOVA compared with P-AH320-PB2_*SH14*_ or P-WT-AH320 virus (^∗^*P* < 0.05; ^∗∗^*P* < 0.01; ^∗∗∗^*P* < 0.001).
