# Supplementary figures and images for: The Packaging Regions of G1-Like PB2 Gene Contribute to Improving the Survival Advantage of Genotype S H9N2 Virus in China
Source: Front Microbiol. 2021 Apr 21;12:655057. doi: 10.3389/fmicb.2021.655057 (PMC8096984; doi:10.3389/fmicb.2021.655057)

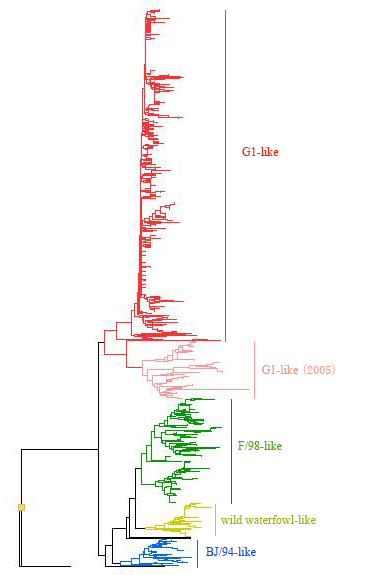

Supplement: Supplementary Figure 1 — Phylogenetic trees of PB2 gene segments of the H9N2 viruses during 1996–2019 isolated in China. [file Image_1.TIF]

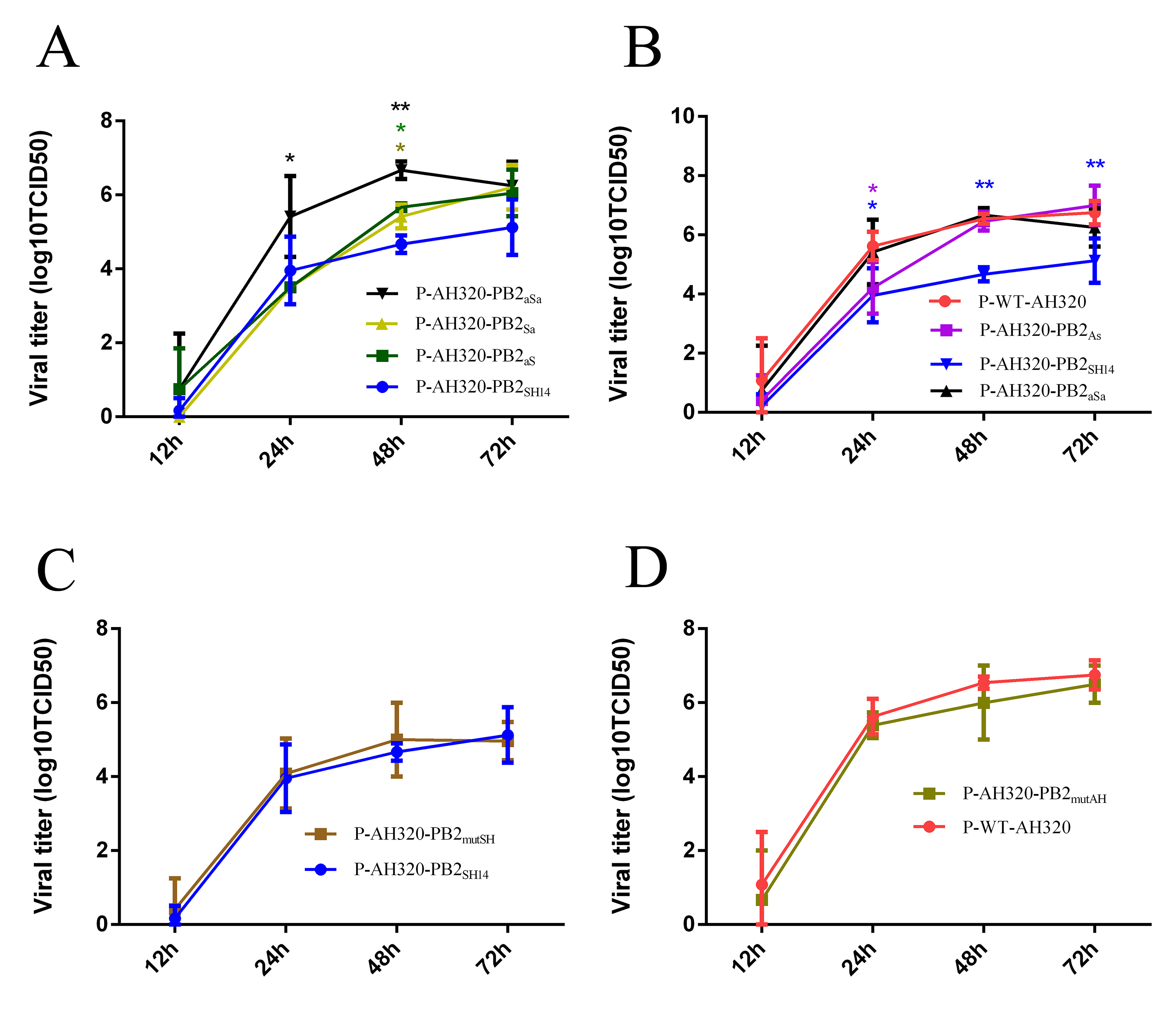

Supplement: Supplementary Figure 2 — Multiple-cycle growth curves of plaque purified H9N2 viruses in CEF cells. The replication abilities of each virus in CEF cells was measured by infecting cells at an MOI of 0.1. Virus titers in supernatant were determined in CEF cells at indicated time points. Data are represented as mean ± SD (N = 3). The statistically significant differences were analyzed by ANOVA compared with P-AH320-PB2SH14 or P-WT-AH320 virus (∗P < 0.05; ∗∗P < 0.01; ∗∗∗P < 0.001). [file Image_2.TIF]
