# Supplementary material for: The Packaging Regions of G1-Like PB2 Gene Contribute to Improving the Survival Advantage of Genotype S H9N2 Virus in China
Source: Front Microbiol. 2021 Apr 21;12:655057. doi: 10.3389/fmicb.2021.655057 (PMC8096984; doi:10.3389/fmicb.2021.655057)
Supplement: Supplementary file 4 [file Data_Sheet_1.pdf]

Table S1. Distance Estimation of PB2 nucleotide sequence (black) and amino acid (red) sequence

Years of isolation	PB2	G1-like	F98-like	Wild waterfowl-like	G1-like (2006)
2007-now	G1-like				
1998-2012	F98-like	0.101/0.022			
-2005	Wild waterfowl-like	0.098/0.023	0.056/0.039		
-2005	G1-like (2006)	0.096/0.052	0.12/0.068	0.118/0.062	
-2006	BJ94-like	0.097/0.020	0.095/0.022	0.09/0.023	0.128/0.052
